# Postbiotics as novel health-promoting ingredients in functional foods

**DOI:** 10.15171/hpp.2020.02

**Published:** 2020-01-28

**Authors:** Aziz Homayouni Rad, Leili Aghebati Maleki, Hossein Samadi Kafil, Hamideh Fathi Zavoshti, Aamin Abbasi

**Affiliations:** ^1^Department of Food Science and Technology, Nutrition Research Center, Tabriz University of Medical Sciences, Tabriz, Iran; ^2^Department of Immunology, Immunology Research Center, Tabriz University of Medical Sciences, Tabriz, Iran; ^3^Department of Microbiology, Drug Applied Research Center, Tabriz University of Medical Sciences, Tabriz, Iran; ^4^Student’s Research Committee, Tabriz University of Medical Sciences, Tabriz, Iran

## Dear Editor,

Clinical investigations have demonstrated that lifestyle factors, especially diet, are significantly associated with a variety of diseases and the host’s health status. In this regard, functional foods containing health-promoting ingredients such as protein, carbohydrate, lipids, vitamins, minerals, phenolic and bioactive components (probiotics, prebiotics, synbiotics and postbiotics) have been attaining more significance by researchers, producers and consumers.^1,2^ These functional foods can be categorized as natural, transformed, fortified, enhanced or enriched foods. A functional food with an ingredient of bioactive components such as probiotics, prebiotics, and postbiotics is considering as an enriched food.^3^ Probiotics are the most investigated and applied components in functional food supplements. Probiotics are characterized as non-pathogenic microorganisms that exert beneficial effects on the host when consumed in sufficient quantities.^4^ Some of the recognized health effects for probiotic supplements includes immunomodulation, anticarcinogenic, effectiveness against diarrhea, antidiabetic, hypolipidemic, and improvement of lactose intolerance.^5-7^ Prebiotics are characterized as non-digestible constituents of functional foods (inulin, oligofructose, stachyose, oligosaccharides, and raffinose) that not affected by human digestive enzymes nonetheless it is fermented via colonized probiotics in the large colon and promoting the colonization of beneficial microbiota and subsequently improving the health of the host.^8^ The main health-promoting effect of prebiotic supplements on the host are decreased cancer risk, balanced cholesterol levels, increased mineral absorption, promote hormonal balance, lipid regulation, lower risk for cardiovascular disease, decreased acute gastroenteritis and lower autoimmune reactions.^9^ Along with increasing consumer awareness, to optimize the positive health effects of probiotics, functional foods containing postbiotic compounds were introduced. The term postbiotics (Nonbiotics) refers to all products obtained from non-viable probiotic microorganisms including non-viable microbial cells, cell walls, lysates, fractions, secretions, components and metabolites that when received in adequate quantities (postbiotic supplements),^10^ endowment healthiness to the host like live probiotic cells.^11^ The advantages of postbiotics in terms of safety, biological and pharmaceutical properties in comparison with live probiotics, includes no risk of translocation from gut lumen to blood, suitable absorption, metabolism, distribution, and excretion potencies, signaling to various organs and tissues in the host and making several biological responses, higher stability and easier to standardize and carrying.^12^ On the other hand, postbiotics used in a delivery system reinforce the endogenous probiotics of each host instead of adding unfamiliar probiotic strains to the gut microbial ecosystem^13^ that can be consider as a safe alternative for live probiotic microbes and applied in functional foods and pharmaceutical industry for creating and developing health benefits, preventing of diseases and therapeutic aims. Additional metabolomics studies are required for the description of novel postbiotic components and survey their safety and constancy during the production processes, marketplace, and host’s digestive system conditions.

## Ethical approval

Not applicable.

## Competing interests

The authors declare that they have no competing interests.

## Authors’ contributions

All authors had active participation in preparation of manuscript.
